# Qualitative Evaluation of Family Caregivers’ Experiences Participating in Knowledge and Interpersonal Skills to Develop Exemplary Relationships (KINDER): Web-Based Intervention to Improve Relationship Quality

**DOI:** 10.2196/42561

**Published:** 2023-08-22

**Authors:** Kylie Meyer, Alexander Gonzalez, Donna Benton

**Affiliations:** 1 Frances Payne Bolton School of Nursing Case Western Reserve University Cleveland, OH United States; 2 Leonard Davis School of Gerontology University of Southern California Los Angeles, CA United States

**Keywords:** aging, Alzheimer’s, Alzheimer, caregiver, caregiving, dementia: digital health, digital intervention, family care, informal care, intervention, older adult, quality of care

## Abstract

**Background:**

The onset of Alzheimer disease and related dementias (AD/ADRD) can alter relationships between family caregivers and persons living with AD/ADRD, such as through the occurrence of distressful behavioral and psychological symptoms of dementia. Poorly perceived relationship quality by caregivers contributes to negative outcomes for both care partners, such as low-quality caregiving and potential mistreatment of older adults. Knowledge and Interpersonal Skills to Develop Exemplary Relationships (KINDER) is a new, web-based, asynchronous psychoeducational intervention with content informed by focus groups with family caregivers. The program was developed to prevent low-quality caregiving and potential mistreatment of older adults by focusing on building healthy caregiving relationships.

**Objective:**

The purpose of this study is to describe caregivers’ experiences participating in KINDER to understand intervention acceptability. Of particular interest was learning how comfortable caregivers were viewing content addressing potential mistreatment, as well as whether asynchronous delivery created any barriers to participating in the intervention. Findings will inform future program refinements before efficacy testing.

**Methods:**

Although 23 caregivers enrolled in the KINDER parent study, only 7 of them completed the 8-week intervention. In-depth, semistructured qualitative interviews were conducted with all participants who completed the program to understand their experiences while attending KINDER and to decipher barriers to participation. We also asked participants about which program elements were most valuable and which were least valuable to them, as well as how the program could be improved. Interview transcripts were analyzed by 2 coders using thematic analysis.

**Results:**

Our findings indicate that caregivers were overall satisfied with KINDER’s focus and content. Participants particularly liked how KINDER materials felt authentic and relevant to supporting healthy care relationships (Theme 1). The program’s multiple components were found to be valuable, especially story-based video vignettes and readings (Theme 2). Most caregivers were comfortable viewing depictions of mistreatment and understood the importance of this content (Theme 3). Notably, while caregivers appreciated the convenience of participating in an asynchronous web-based intervention, several expressed a desire for more opportunities to speak with other caregivers (Theme 4). Technology challenges, such as a lack of clarity about automated intervention activities, deterred completion.

**Conclusions:**

Findings from this study suggest an asynchronous web-based intervention covering sensitive topics such as mistreatment is acceptable for at least some AD/ADRD caregivers. Caregivers’ comments that materials felt authentic may suggest that the integration of caregiver voices before intervention development enhanced the relevance of content. To make KINDER easier to deliver and participate in, the investigators plan to reduce the use of automation and integrate more group-based programming, as recommended by participants. Further, given the higher-than-expected dropout rate, in future studies, the investigators will collect data to determine the reasons for participants not completing study activities.

## Introduction

### Overview

In the United States, an estimated 11 million family caregivers provide the majority of care to persons living with Alzheimer disease and related dementias (AD/ADRD) [1]. Caregivers help persons living with AD/ADRD complete instrumental tasks, such as help with shopping and transportation, as well as more intensive personal care, including bathing and grooming. AD/ADRD caregiving is distinguished from other types of family caregiving by the presence of behavioral and psychological symptoms of dementia (BPSD), which affect approximately 9 out of 10 persons living with AD/ADRD [2]. BPSD can be highly distressing to caregivers and worsen mental health [3,4]. BPSD can also increase the risk that caregivers will provide low-quality care, including mistreatment (older adult mistreatment) [5,6]. While multiple interventions exist to help caregivers reduce stress and manage BPSD [7], few have targeted the caregiving relationship directly.

### Caregiving Relationship Quality and Quality of Care

Relationship quality (RQ) between caregivers and persons living with AD/ADRD contributes to both partners’ health and may be an underused intermediate intervention target to improve outcomes for both care partners. RQ within caregiving refers to various aspects of the care relationship, including satisfaction, positive interactions, and emotional support, and is largely subjective [8]. RQ may be diminished following the onset of AD/ADRD [8]. Caregivers describe how BPSD in their care recipient can erode the dyad relationship as it limits the person living with AD/ADRD’s ability to communicate and show affection [3]. RQ also moderates caregivers’ negative appraisals of BPSD; while BPSD are a cause of caregiver distress, this association is less pronounced among caregivers who report higher RQ [9]. RQ is also posited to affect the quality of care provided by family members, including the likelihood of providing potentially harmful care and mistreatment [10,11].

### Interventions to Support Caregiving Relationship Quality

Few intervention studies examine relationship quality as a primary outcome in AD/ADRD caregiving, though several consider RQ a secondary outcome or include content that supports a relational approach to caregiving (eg, building empathy) [7]. In the Through the D’mentia Lens intervention by Wijma et al [12], researchers administered an internet-based intervention to improve caregivers’ understanding of what it is like to live with dementia. Findings from a pre-and posttest evaluation demonstrate improvements in positive interactions between care partners, according to the caregivers. In another study, researchers examined whether activity-focused content (ie, behavioral activation, such as planning a pleasurable activity) provided additional benefits beyond AD/ADRD education alone to improve RQ [13]. Findings from this randomized controlled trial showed that, while both groups reported improved relationship satisfaction, caregivers who also completed behavioral activation telephone consultations demonstrated additional improvements compared to those who completed AD/ADRD education alone.

Findings from these 2 studies suggest 2 features that are likely important to RQ interventions designed for AD/ADRD caregivers. First, AD/ADRD education is important to help improve caregiver understanding of the disease, as suggested by basic behavioral studies [14]. Second, the integration of psychoeducational components, rather than education alone, improves intervention efficacy. This finding is consistent with previous studies that support the superiority of psychoeducational caregiver interventions over education alone [15].

Illustrating the importance of psychoeducation to improve RQ in another population is the ePREP intervention to support healthy intimate partner relationships [16]. This self-paced intervention includes videos, readings, and assignments that are delivered on the web and can be completed in participants’ homes. While not developed to prevent intimate partner violence, participation in ePREP is associated with a reduction in situational intimate partner violence reported by individuals and their partners, in addition to improvements in RQ [17]. The ability to engage with sensitive content, such as about relationship tension and potential mistreatment, in a private setting may also be advantageous to AD/ADRD caregiver RQ interventions. In a study of an in-person music therapy program for both AD/ADRD care partners, researchers discovered a ceiling effect for change in RQ as well as low uptake among care partners who initially expressed interest [18]. These findings suggest that caregivers who could most benefit from an RQ caregiving intervention (ie, those beginning with low RQ) may prefer a self-administered approach.

### Purpose of This Study

Based on findings from previous interventions to improve RQ between AD/ADRD care partners and findings from the ePREP intervention, the researchers developed a web-based, multicomponent psychoeducational intervention to improve caregiving RQ and quality of care, including the prevention of mistreatment of older adults. The resulting program was named Knowledge and Interpersonal Skills to Develop Exemplary Relationships (KINDER). The purpose of this initial pilot study was to learn about (1) caregivers’ experiences participating in the KINDER intervention and (2) opportunities to improve the delivery of content to support future efficacy testing.

Findings from this study are intended to inform intervention acceptability, as defined by Sekhon et al [19]. Based on findings from their systematic review, they define “acceptability” as the extent to which those who receive an intervention believe it to be appropriate based on either their anticipated or experienced responses. In their Theoretical Framework of Acceptability (TFA), these authors further propose 7 domains of acceptability, including participants’ affective attitude about the intervention, perceived burden of participation, alignment with values (ethicality), perceived intervention coherence, opportunity costs to participants (eg, is participating in the program worth the time and effort required), perceived effectiveness, and participant confidence they can perform the required behaviors to complete the intervention (self-efficacy). To assess the anticipated acceptability of an intervention during the pilot phase, Sekhon et al [19] recommend using semistructured interviews. Accordingly, we conducted in-depth qualitative interviews with caregivers who participated in the KINDER program.

## Methods

### Overview

The researchers conducted 7 semi-structured, one-on-one qualitative interviews on Zoom with family caregivers who participated in the KINDER intervention from March 2020 to September 2021. This study was conducted as a part of a parent study, which entailed a single-arm pre- and posttest design trial. This study focuses on the qualitative interviews conducted after caregivers participated in KINDER.

### Intervention

KINDER includes 8 weekly lessons that cover information about AD/ADRD, navigation of community-based resources, family relationships, strategies for communication, ways of coping, self-care, and mental health. Topics for each lesson were based on previous research regarding factors affecting care relationships and care quality, as well as focus groups with family caregivers about how they cope with relationship tension [20,21]. To support the cultural relevance of KINDER during its development, focus groups were conducted with a racially and ethnically diverse sample of caregivers, including 2 Latino, 2 African American, 2 Asian, 1 White, and 1 mixed race or ethnicity group. The focus groups were conducted in English, Spanish, and Mandarin, according to the participant’s first language. Intervention materials were designed to reflect caregivers from multiple identities, including race and ethnicity, age, gender, sexual orientation, and socioeconomic status.

Each KINDER lesson began with a 3- to 5-minute story-based video, followed by reading content that complemented the issues presented in the video and a short, 3- to 5-question reading quiz. Caregivers were also encouraged to generate and track progress on a self-care goal each week and to complete weekly reflection exercises pertaining to lesson content. All study materials were delivered over a web-based platform using a unique login, and participants received an email reminder when a new lesson was available. In the parent study, the same web-based platform used to deliver KINDER was also used to securely collect self-administered surveys. Images of the website and an example video vignette can be found in Multimedia Appendix 1.

### Eligibility

To be eligible to participate in the KINDER parent study and, thus, this substudy, participants had to be caregivers to a person living with dementia aged 18 years or older and provide care for at least 8 hours a day. Caregivers had to help with at least 2 instrumental activities of daily living or 1 activity of daily living. Exclusion criteria included being unable to read or speak English and the inability to participate in a telephone or Zoom interview. Further, to be asked to participate in the interview, caregivers were required to have completed the KINDER program. Program completion was required so that participants could sufficiently comment about the value of program components and the content presented in all lessons.

### Recruitment

Recruitment for the KINDER parent study primarily occurred at an academic service center in Southern California that regularly provides caregiver education, assessment, information, referral, and care planning. Caregivers could learn about the parent study through newsletters, social media posts, or through their designated care navigator. For this qualitative interview substudy, participants who completed KINDER were emailed by the program administrator to ask whether they were interested in completing a qualitative interview. If they agreed, the participant was introduced to the researcher conducting the qualitative interviews.

### Data Collection

One-on-one, semistructured, 1-time Zoom interviews were administered by the first author, a PhD in Gerontology who was a postdoctoral researcher at the time of data collection. This researcher had graduate-level training in qualitative methods as well as multiple publications using interview methods. She was not involved in administering the intervention to caregivers to prevent the likelihood that caregivers would avoid criticizing the KINDER intervention when speaking with a program facilitator. Caregivers knew from study recruitment emails and the information sheet that the interviewer was affiliated with a different institute than the institute where the parent study occurred. Interview questions were asked about caregivers’ experiences participating in the KINDER program, such as what they learned, as well as their recommendations to improve the intervention. Textbox 1 lists the interview topics, and Multimedia Appendix 2 provides the interview guide. Interviews took approximately 30 minutes to complete, and all were video recorded and transcribed verbatim.

**Interview topics.**

**Topics included in the interviews:**

- How caregivers thought Knowledge and Interpersonal Skills to Develop Exemplary Relationships (KINDER) could affect caregiving relationships
- The most and least valuable components of the KINDER intervention for supporting caregiving relationships
- Who do caregivers think would most benefit from participation in KINDER
- Caregivers’ feelings of discomfort while participating in KINDER, such as content pertaining to mistreatment
- Barriers to participating in KINDER intervention, including technology errors
- Recommendations to improve KINDER for future caregivers

### Analysis

We used a thematic analysis to analyze the interviews [22]. Transcripts were analyzed by 2 independent coders. Before beginning coding, these researchers read through 2 transcripts to create an initial codebook. Codes were developed to address topics discussed in the interview guide, though coders also considered inductively derived codes. Using this codebook, they coded transcripts independently and then met to review the coding together each week. Coding discrepancies were discussed until agreement was reached. New codes were added to the codebook during the first round of coding as needed. Before conducting a second round of coding, a third researcher reviewed all code categories to ensure consistent application of codes to text excerpts to enhance the trustworthiness of findings [23]. Code definitions were refined accordingly, and affected codes were reviewed in a second round of coding, during which new codes were also added to previously coded transcripts. An abbreviated codebook can be found in Multimedia Appendix 3. Analyses were conducted in NVivo 12 (QSR International).

### Ethical Considerations

This research was reviewed and given an “exempt” determination by the University of Texas Health Science Center (HSC20200569E) and Case Western Reserve University (STUDY20220984). All participants provided verbal consent before participating in this research and received a copy of the study information sheet by email. Data were deidentified before being analyzed. No payment was offered for participating in the interview, but it was provided for the parent study. The parent study is registered at ClinicalTrials.gov (NCT03593564). Multimedia Appendix 4 includes the consolidated criteria for reporting qualitative research for this study [24].

## Results

### Overview

From March 2020 to September 2021, a total of 23 caregivers were found to be eligible and consented to participate in the KINDER study. Of those caregivers, 7 completed the program. The 7 participants who completed the program were invited to complete an interview, and all agreed. Participant characteristics can be found in Table 1.

**Table 1:** Participant characteristics.

Participant number	Age (years)	Gender	Race and ethnicity	Educational attainment	Number of years caregiving	Care recipient relationship to caregiver
1	71	Female	Black or African American	Some college	17	Husband
4	58	Female	Non-Hispanic White	Some college	16	Mother and Father
5	60	Female	Hispanic or Latino	Some college	5	Mother
6	80	Female	Indonesian	Some college	5	Husband
7	60	Female	Black or African American	Some college	3	Husband
8	55	Female	Black or African American	College degree	8	Mother
9	Missing	Female	Black or African American	Graduate degree	3	Father

### Themes From Qualitative Interviews

#### Theme 1: Caregivers Found KINDER to be Authentic and Relevant to Supporting Healthy Care Relationships

Overall, caregivers reported satisfaction with the KINDER program. “I just love this program,” said one caregiver.

It resonated with me and it’s something that I will continue to use and talk about to others.

Participant 5

Echoing this enthusiasm, another interview participant said of KINDER, “I think it’s a game changer in our lives. I really do” (Participant 8).

Other caregivers shared how KINDER’s relationship-focused approach caught their interest:

So just even the thought of the program made me feel as though it was going to help me ease into the challenge of caregiving in a more natural way and in a kinder way, and a more caring, authentic way, as opposed to something that is obligatory...

Participant 9

This authenticity also appeared to facilitate caregivers’ openness to learning about managing challenging aspects of the caregiving relationship. A caregiver assisting both of her parents shared how the program addressed some of the under-recognized aspects of caregiving, including how difficult it is:

...if I had a penny for every person that told me, “You’re so lucky to still have your parents” you know? Yes, I am. It’s a lot of work and it’s not easy. Of course, I do it with the kindness of my heart, but sometimes it’s nice when someone can understand you and just listen and say, “Yeah, I know. I know what you mean... or “I went through it too.” And that was a thing with KINDER, it’s like you felt that connection. This person, whoever did that, knew... and you felt that connection, and, as a caregiver, you like that connection.

Participant 4

These comments suggest that KINDER was relatable and felt true to caregivers’ lived experiences.

Caregivers also found KINDER to be relevant to supporting their caregiving relationship. When asked whether she thought that KINDER could support a healthy caregiving relationship, a spousal caregiver said, “Gosh, I think it would improve that relationship and maybe strengthen it...” (Participant 7).

Elaborating on this point, another caregiver shared how the self-care information within the KINDER intervention helped her to act more compassionately toward her loved one:

You inadvertently, subconsciously became a kinder caregiver... because now the things that were taking away the energy that you need to be compassionate—those were the barriers that were decreasing.

Participant 9

This comment reflects a sentiment emphasized throughout the KINDER intervention that “you cannot pour from an empty cup,” an expression the study team learned from a focus group participant and included in the intervention.

#### Theme 2: Caregivers Valued KINDER’s Multicomponent Approach to Asynchronous Delivery

One of the anticipated risks of the KINDER intervention was that caregivers would not find the program sufficiently engaging, given the reliance on an asynchronous modality of delivery. The investigators wanted to ensure that each component of KINDER was valuable to the caregivers since caregivers have limited time to spend on interventions given the competing demands on their time. Responses to questions about how much caregivers valued each component of KINDER revealed that caregivers, overall, found value in each component.

#### Video Vignettes

One of the most valued parts of the KINDER program was the video vignettes at the start of each lesson. A spousal caregiver said:

The videos were excellent because they gave you an idea of what the chapter was going to be, and everything in the video was emphasized in the reading.

Participant 1

Participants emphasized how relatable this content was:

I was impressed because I would watch the videos over and over again, and they’re relatable. These people were relatable.

Participant 5

Echoing this, another caregiver said:

I love how there was always a video, and I could relate to every video that there was.

Participant 4

The investigators also learned that caregivers would need to return to the videos to rewatch them after reviewing the rest of the lesson.

I watched them, maybe impatiently isn’t the word... and I saw them, but I think it wasn’t until I digested the information—I kind of go back, and I’m like, watching then.

Participant 9

Together, these comments suggest that while videos may have caught caregivers’ interest, caregivers may need time and support to make sense of the content.

#### Readings

Caregivers also valued the written information that elaborated on the topics raised in the videos. Although several caregivers felt “there was a lot of text,” it was largely recognized that all the information provided was important (Participant 8).

“I noticed that like every sentence, it doesn’t feel like anything is extra,” said one caregiver, who also found KINDER readings to be text-heavy, “And so with that said, like I don’t want to miss anything” (Participant 9).

Integrated within the KINDER text were resources where caregivers could explore topics addressed in KINDER in even greater depth. The caregivers particularly liked having access to these resources. One caregiver said:

And these were good, solid links. Some of them or several I saved or I wrote down a lot of things.

Participant 4

Another caregiver said, “I pretty much clicked on every link to every website” (Participant 7). One said these links made her feel like “I was getting the latest information which, again, is super important” (Participant 5).

#### Reading Quizzes, Reflections, and Goal-Setting

Caregivers also recognized the value of the exercises that accompanied the videos and text but identified areas for improvement. At the end of each reading, there was a quiz to help the caregivers check their knowledge of the content. While most of them did not comment on the quizzes, those who did described them in positive terms: “These are helpful quizzes. I don’t feel stupid. I feel encouraged. It’s very positive” (Participant 5). This caregiver emphasized the use of positive prompts to guide caregivers to the correct answers and positive reinforcement when they selected the correct response. She also reported positive experiences with the reflection exercises:

These are wonderful prompts for reflection. It was fantastic to help me then go into deep meditation and breathing, and delve in deeper and let go, let go, let go, let go, and find what the truth is.

Participant 5

Still, most participants did not comment on the reflection exercises, which may similarly indicate a lack of engagement in this asynchronous activity.

Caregivers were less positive about the goal-setting feature. Although many liked the idea of setting self-care goals, multiple caregivers noted that this feature was not intuitive. When asked about her experience with the goal-setting feature, one caregiver said:

Okay, my first reaction is to say, you have this really awesome idea... And listen, I love a good list and vision boards and things like that. But I think the goal setting, something in the way it was designed, wasn’t as helpful to me.

Participant 9

This caregiver later said she felt unclear about how to use the goal-setting feature, such as when to create new goals. These comments suggest that while caregivers might value a goal-setting feature, this activity could have been better designed.

#### Theme 3: Caregivers Felt Comfortable Watching Depictions of Mistreatment

Given the risk of low-quality care and potential mistreatment consequent to low-quality relationships, the investigator team included content about potential verbal mistreatment and neglect in KINDER. All except 1 caregiver reported that they felt comfortable with materials focused on low-quality care and mistreatment. One caregiver referred specifically to a lesson where mistreatment was most directly addressed, which included a video portrayal of a caregiver yelling at the care recipient and using intimidating body language.

It was even, well [lesson] six and seven that you find yourself becoming so angry and so frustrated that you just feel like you’re at your wit’s end. That’s stressful. It’s hard to, if you haven’t been there, to think that you might even go there because of what you’re dealing with.

Participant 7

Most caregivers did not indicate relating to this content directly but affirmed the importance of including it; as 1 caregiver said, “Because you know, unfortunately, things happen.” She continued, “... so I didn’t feel bothered” (Participant 8). Another participant said she was “glad it was in there because maybe it’ll trigger something for somebody” (Participant 4). Still, 1 caregiver did report feeling somewhat uncomfortable with portrayals of mistreatment. “The anger, I think, made me feel uneasy” (Participant 6). She said during other parts of the interview that she could not relate to this experience, and thus this program content may not have resonated with her the way it did with other caregivers.

#### Theme 4: Caregivers Appreciated the Flexibility of the Asynchronous Program but Wished to Speak With Other Caregivers

The reason for building KINDER as an asynchronous program was to make it easier for family caregivers to participate despite busy schedules. Caregivers agreed that the ability to complete lessons at any time was an important feature of the program. One participant said:

You can do it from home at your own pace, like fast-forwarding or going back, even with the book.

Participant 1

Given this flexibility, caregivers could complete lessons when their loved one was asleep, as 1 remarked, “the best time for me to do things like that is late at night, and so that’s when I would do it...” (Participant 4).

Even though caregivers appreciated the flexibility with which they could complete lessons, they also indicated a desire to interact with other caregivers. “There should be group sessions, maybe that people will at some point finally get together and talk,” said 1 caregiver (Participant 5).

It’d be nice if we did have some sort of ongoing voluntary support group or something, so we could sit and talk about these lessons a little—maybe that would help the ‘digestion process,’ although, she admitted, Who has time?

Participant 9

To address this concern, caregivers suggested ideas such as using a Facebook group or small group meetings with 2 to 3 caregivers that could accommodate scheduling challenges. Another suggestion was to support access to a one-on-one counselor to help caregivers discuss content from each lesson that they wished to discuss further.

#### Other Recommendations to Improve KINDER

The research team was also interested in learning how to improve the KINDER program in future iterations. Caregivers shared multiple ways in which KINDER could be improved.

##### Target Family Caregivers New to This Role

Multiple participants shared that KINDER was most appropriate for those who were new to the caregiving role:

I think it would be good for people who are just starting out on their caregiving journey. It would give them a good overview or perspective of some of the things they might encounter.

Participant 7

Another participant echoed the idea: “this was the kind of thing I would give to someone who was a brand-new caregiver and had no idea what they were doing” (Participant 9).

##### Help Caregivers Manage the Large Amount of Content

Caregivers also commented about the amount of content covered in KINDER, and how they felt pressed for time to complete each lesson. One caregiver described her experience:

What would happen was I needed more time with that week’s lesson to digest it and to spend some time really processing it cognitively, emotionally.

Participant 9

To address this, she recommended allowing caregivers more time to complete each lesson at their own pace. Another caregiver also suggested making lessons “briefer, because people now are on the go with technology in their hands and are in a hurry” (Participant 1).

##### Address Quality Issues

Finally, caregivers mentioned several issues related to quality, such as broken links and typographical errors in the text. One said, “I came across a number of grammar errors” (Participant 9). Several caregivers also ran into technology challenges, wherein they received the posttest assessment before finishing lessons since assessments were sent automatically when participants were due to complete lessons.

## Discussion

### Overview

Overall, the caregivers who participated in the KINDER web-based intervention focused on caregiving relationships found it to be a valuable resource. They were particularly fond of the story-based videos, which felt authentic and helped them realize they were not alone in their experiences. Although the content was well liked, there were multiple challenges with delivery and presentation, such as not understanding how to use automated program features. We now consider how caregivers’ experiences convey KINDER’s acceptability, including how findings relate to domains within Sekhon’s TFA [19].

### Comparison of Findings With Previous Research

Findings from this study reiterate the value of AD/ADRD education as a component of RQ interventions, as found in previous studies [12,13]. We also learned from caregivers that the program may be particularly well-suited for caregivers of persons recently diagnosed with AD/ADRD who may not yet understand its symptoms, such as behavioral and psychological symptoms of dementia. Within the TFA model, focusing on delivering KINDER to newer caregivers may improve the perceived value or opportunity costs for participants [19]. These remarks are also consistent with findings from observational studies of AD/ADRD care partners, where caregivers describe adjusting expectations as they learned more about the disease, including behavioral symptoms [20,25]. Poor disease knowledge is posited to contribute to biased perceptions of care recipients and emotional reactivity that can adversely affect the care relationship [26]. KINDER may help newer caregivers cope more quickly with relationship changes than they might otherwise. We plan to test this possibility in a future study.

Caregivers also said they felt KINDER content was authentic, especially the story-based videos. Within the TFA, this indicates meeting the “affective attitude” component of acceptability, or how participants felt about the intervention [19]. Participants reported feeling less alone in experiencing negative thoughts and feelings about caregiving, and even about the care recipient. This sense of authenticity may be attributed to the integration of early stakeholder input. Integration of stakeholder input to develop intervention content was previously found to improve the relevance of RQ intervention in other caregiving populations [27]. The integration of coping mechanisms recommended by caregivers themselves in focus groups is aligned with experiential approaches to intervention development and may improve intervention acceptability [28].

We also learned that most caregivers were comfortable with content portraying and describing poor-quality care and potential mistreatment (eg, a caregiver yelling at a care recipient). Within the theoretical model of acceptability, this finding suggests that KINDER likely demonstrates ethicality or alignment with caregivers’ value system [19]. This is an important finding since low-quality care is posited to be a secondary outcome of low RQ. [10,11] Our finding that caregivers felt comfortable with this content is consistent with previous research showing caregivers frequently self-report mistreatment behaviors in clinic settings, particularly psychological mistreatment [29]. The use of a self-administered web-based intervention may encourage participation among caregivers who might otherwise choose to participate in an intervention to prevent low-quality care and mistreatment.

At the same time, while caregivers interviewed in this study valued self-administration, several indicated they would like the chance to talk to other caregivers. Given that data were collected during phases of the COVID-19 pandemic, it is possible that caregivers were particularly eager to socialize with others [30]. Regardless of this possible biasing factor, group-based psychoeducation has multiple advantages over individual intervention, such as allowing caregivers to learn from one another and to provide peer encouragement [31].

A recent combination synchronous or asynchronous randomized controlled trial of the Tele-Savvy intervention by Hepburn et al [32] demonstrated efficacy at improving caregiver depression, suggesting a “hybrid” option is a promising delivery approach. As such, future iterations of KINDER will include facilitated group discussions to complement the asynchronous components. Integration of discussion sessions also presents an opportunity to reduce text-based information that was found to be too detailed for some participants. Participants who need more time to digest content, such as story-based videos, may also appreciate the opportunity to discuss content in a group setting. While group-based sessions may increase participant burden in some cases given additional time constraints, this may be a worthwhile tradeoff to improve perceived intervention effectiveness within the TFA model [19].

### Improving Future Intervention Delivery

Consistent with previous research on digital interventions among caregivers, web-based delivery of the program also appeared to be well-liked and allowed caregivers to complete activities at their convenience [33,34]. Multicomponent, self-administered, web-based interventions have previously been shown to improve depression and anxiety among AD/ADRD caregivers and may have broader applications in the future [35]. Our findings support the acceptability of web-based intervention delivery to caregivers but also reveal opportunities to improve acceptability, such as by integrating group-based discussion sessions.

Although the purpose of this study was not to assess technology performance, given its effect on participant experiences and its relevance to future researchers, we share how multiple technology issues may have undermined participant completion rates. Because the development of the KINDER program took longer than expected, the study team only completed cursory beta testing of the system and study protocols. We would highly recommend future researchers allocate enough time in their grant timelines to allow for thorough testing of web-based interventions and study protocols. (eg, 2-3 months) [36]. The application of a digital intervention checklist, as proposed by Bartlett Ellis et al [37] could also prevent the challenges this study team encountered. Previous studies have also found technology issues to be a distraction to participants in AD/ADRD caregiving interventions and undermine program engagement [38].

### Study Limitations

Although generalizability is not a relevant criterion with which to evaluate qualitative studies, it is important to address the likely effects of sample selection on this study’s findings. This study examined the experiences of caregivers who completed KINDER and is not likely to reflect the experiences of those who did not complete the intervention. Indeed, although 23 caregivers enrolled in KINDER, only 7 completed the program. While we believe that attrition may be due to challenges with the intervention delivery mode, including technology issues and the desire to engage with other caregivers, this cannot be certain. An alternative cause may be caregivers’ motivation to participate in an intervention focused on the caregiving relationship. Supporting this possibility are similar findings of high attrition by Tamplin et al [18] in their music intervention to support relationships among family caregivers and persons living with AD/ADRD. In the next phase of this research, the investigators plan to address this limitation by collecting survey information about participants’ reasons for withdrawing from the study or intervention using response categories used by the National Institute on Aging’s Clinical Research Operations and Management System (CROMS). Despite these limitations, the authors believe it is important to report on initial findings to help guide researchers focused on relationship-focused caregiver intervention in the future, as well as to provide lessons learned from technological challenges.

### Conclusions

Findings from this qualitative study of the KINDER program support its continued development to assist healthy caregiving relationships toward the goal of preventing low-quality care and potential mistreatment of older adults. Such interventions are important given the impact of changes in the care relationship on both care partners [9,39]. Findings from this study show that caregivers welcomed the focus on the care relationship to approach the topic of care quality. Future research will entail a new pre- and posttest analysis of the KINDER intervention to test its preliminary efficacy, following the completion of improvements indicated by qualitative findings (eg, integration of discussion groups). Ultimately, this research has the potential to help caregivers provide care that promotes the health and well-being of both care partners.

## Appendix

Multimedia Appendix 1 Images of KINDER intervention site, including URL to example video featured in lesson.

Multimedia Appendix 2 Participant Interview Guide.

Multimedia Appendix 3 Abbreviated qualitative codebook used to analyze transcripts.

Multimedia Appendix 4 Consolidated criteria for reporting qualitative research.

## Abbreviations

AD/ADRD: Alzheimer disease and related dementias
BPSD: behavioral and psychological symptoms of dementia
CROMS: Clinical Research Operations and Management System
KINDER: Knowledge and Interpersonal Skills to Develop Exemplary Relationships
RQ: relationship quality
TFA: Theoretical Framework of Acceptability

## Data Availability

The interview data generated during the current study are not available. Given the potential for sensitive information to be discussed during interviews related to the quality of care, the informed consent documentation used in this study did not allow for sharing data outside the research team.
