# Supplementary material for: Qualitative Evaluation of Family Caregivers’ Experiences Participating in Knowledge and Interpersonal Skills to Develop Exemplary Relationships (KINDER): Web-Based Intervention to Improve Relationship Quality
Source: JMIR Form Res. 2023 Aug 22;7:e42561. doi: 10.2196/42561 (PMC10481209; doi:10.2196/42561)
Supplement: Multimedia Appendix 1 [file formative_v7i1e42561_app1.docx]

**Multimedia Appendix 1**

**KINDER Login Page**

**
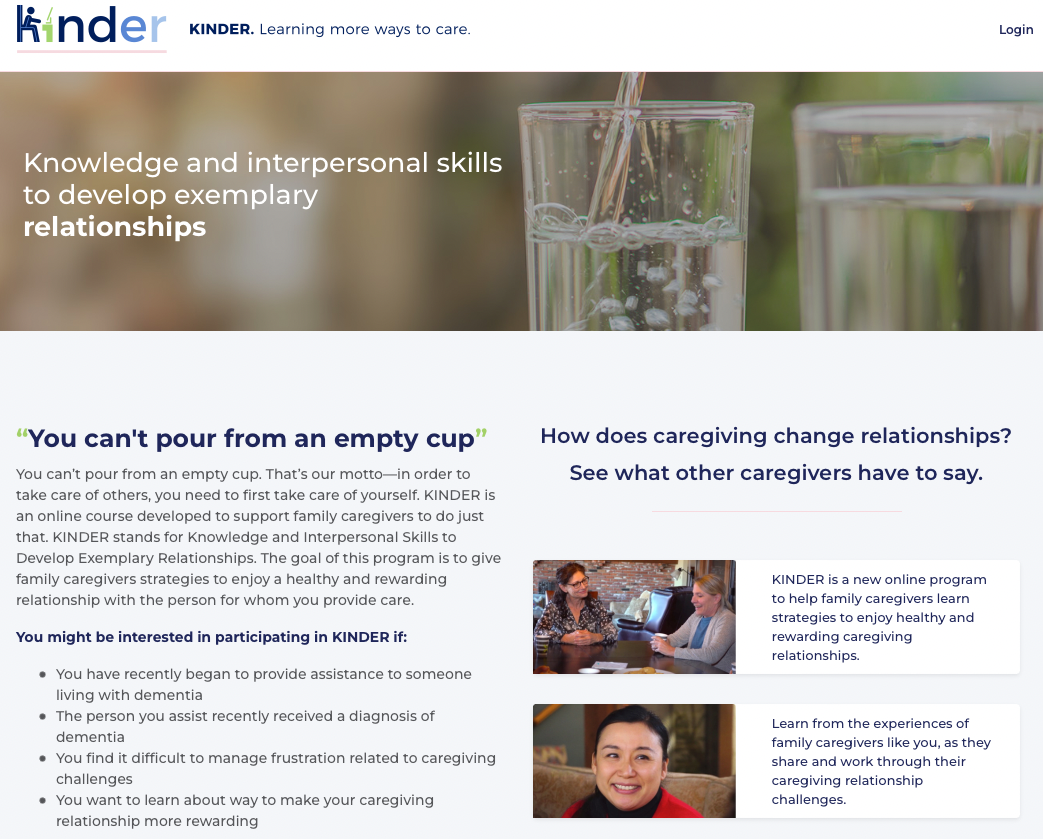
**

**KINDER Lesson Example, Video and Text**

**
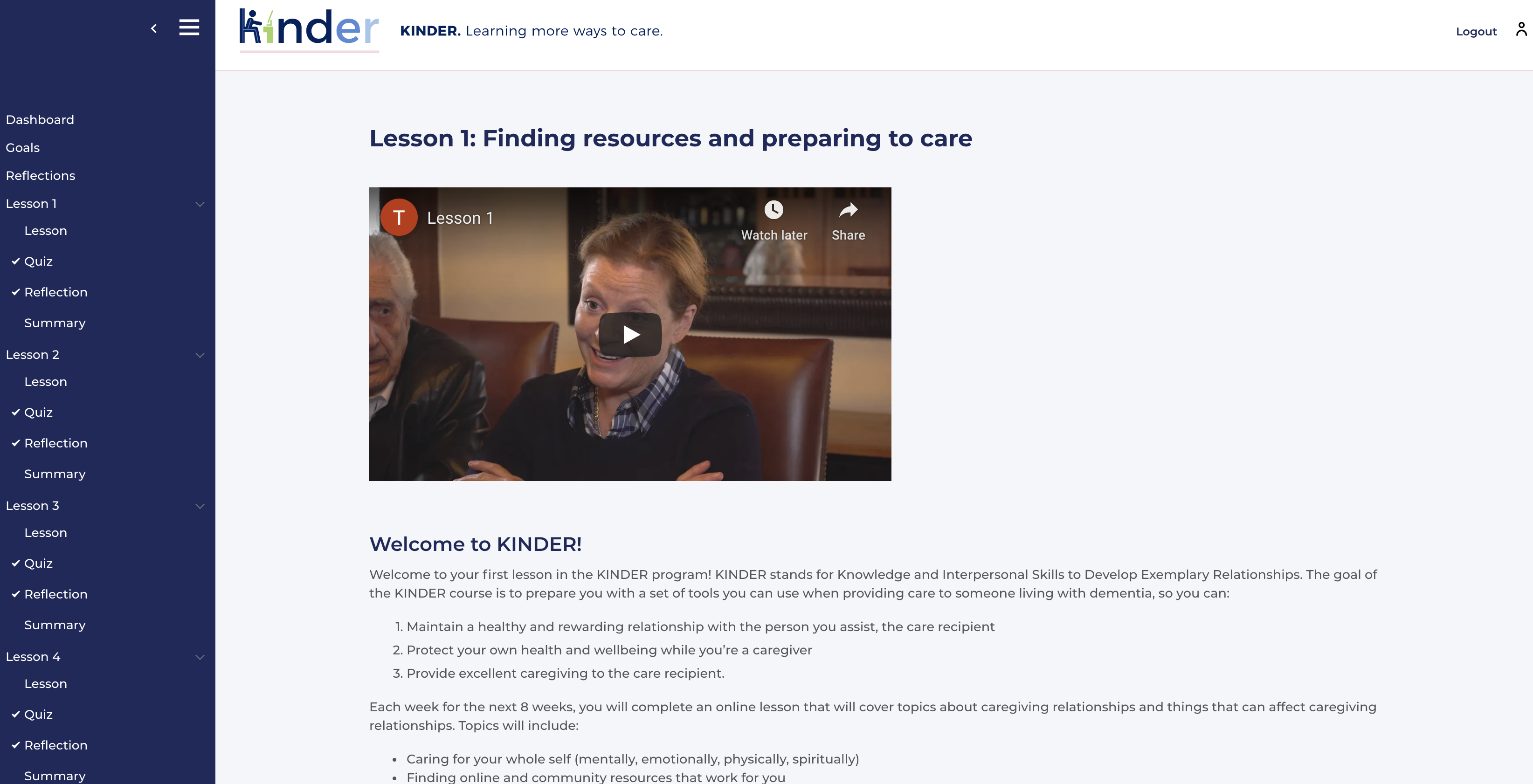
**

**Link to Example Video:**

<https://www.youtube.com/watch?v=1H_Id0djpkA>
