# Supplementary material for: Qualitative Evaluation of Family Caregivers’ Experiences Participating in Knowledge and Interpersonal Skills to Develop Exemplary Relationships (KINDER): Web-Based Intervention to Improve Relationship Quality
Source: JMIR Form Res. 2023 Aug 22;7:e42561. doi: 10.2196/42561 (PMC10481209; doi:10.2196/42561)
Supplement: Multimedia Appendix 2 [file formative_v7i1e42561_app2.docx]

**Multimedia Appendix 2**

**Interview guide for user-experience of the KINDER online intervention program**

1. From your perspective, what do you think was the overall purpose of the KINDER program?

*Following answer, confirm or clarify that the purpose of KINDER is to promote healthy caregiving relationships.*

1. In what ways do you think that the KINDER program may affect caregivers’ relationships with the individuals for whom they are caring? What other benefits do you expect caregivers will experience from participating in the KINDER program?
2. What aspects of the KINDER program do you think would be most impactful in improving caregivers’ relationships with care recipients?
3. What information or components within the program do you think would be least valuable at improving caregivers’ relationships with care recipients?
4. Why do you think a caregiver might consider participating a program like KINDER?
5. Do you think there are some caregivers who would benefit more from the KIDNER program than others? If so, could you describe the kind of caregiver you think could most benefit from participating?
6. Were there parts of the KINDER program that made you feel uncomfortable or uneasy? What were these and why do you think they made you uneasy? (Are there things you recommend we could do to reduce caregiver discomfort?)
7. What aspects of the KINDER program promoted your trust in the information provided? Which aspects of the program made it less trustworthy?
8. Did you experience any barriers to participating in the KINDER program, such as with the technology, the amount of time it took, or any other issues? What were these?
9. What recommendations do you have to improve the KINDER program for future caregivers?
10. Would you recommend the KINDER program to a friend who is providing care for someone living with dementia? Why or why not?
11. Is there anything you would like to tell me about the KINDER program that I have not asked you?
