# Supplementary material for: Qualitative Evaluation of Family Caregivers’ Experiences Participating in Knowledge and Interpersonal Skills to Develop Exemplary Relationships (KINDER): Web-Based Intervention to Improve Relationship Quality
Source: JMIR Form Res. 2023 Aug 22;7:e42561. doi: 10.2196/42561 (PMC10481209; doi:10.2196/42561)
Supplement: Multimedia Appendix 3 [file formative_v7i1e42561_app3.docx]

**Multimedia Appendix 3**

| **Table 3: Codebook** | |
| --- | --- |
| **Code categories and code names** | **Definitions** |
| **Relationship experiences caregivers felt were well-addressed in the program** | |
| Relationship turbulence | Caregiver describes experiencing turbulence in their relationship, such as onset of new conflicts following diagnosis or onset of care, which KINDER addressed |
| Relationship uncertainty | Caregiver describes feeling uncertain in their relationship, such as lack of assuredness about their role or how to support family member living with dementia, which KINDER addressed |
| Relationship history | Caregiver describes relating to content within KINDER about tensions in relationship history with the care recipient |
| Self-identification as caregiver | Caregiver is better able to understand and define their role as a caregiver following participation in the KINDER program |
| It’s not just me | Caregiver describes feeling like they are not alone, and that others are experiencing similar relationship challenges following participation in KINDER |
| **What caregivers like about KINDER** | |
| Could do at any time/flexibility | Caregiver describes liking that they could complete KINDER activities at their convenience |
| Videos/story | Caregiver describes liking the story-based videos |
| Resources | Caregiver describes liking the resources referred to in the KINDER program |
| Readings | Caregiver expresses liking the written content found within KINDER |
| Exercises (reflection, quizzes) | Caregiver expresses liking the activities integrated into the KINDER program, such as quizzes and reflection exercises |
| How did caregivers use KINDER? | |
| Repeat viewings vs. one-time viewings | Caregiver describes frequency of viewing KINDER content: once or multiple times |
| Practiced strategies/took action | Caregiver describes applying lessons from KINDER and/or skills that they’ve learned |
| Completed exercises | Caregiver describes completing KINDER exercises, including quizzes and reflection exercises |
| Extent of engagement with lessons | Caregiver describes the extent to which they engaged with lessons (eg, completed all quizzes, skipped some reading) |
| Timing of completion | Caregiver describes how long it took to complete KINDER, such as quickly moving through lessons or taking their time |
| Sharing with others | Caregiver describes sharing what they learned from the KINDER program with others |
| Other ways of using KINDER | Caregiver describes other ways in which they utilized the KINDER program |
| **Recommendations** | |
| Timing of delivery in care trajectory | Caregiver recommends that KINDER be provided to caregivers at a certain time in the care journey, such as following a diagnosis |
| Group-based sessions | Caregiver expresses an interest in attending group-based sessions with other caregivers |
| Quality of materials | Caregiver comments on the quality of the KINDER materials, such as spelling and grammar issues |
| Technology issues | Caregiver describes experiencing technology-based challenges to participating |
| Other recommendations | Caregiver provides another recommendation other than those listed to improve the KINDER program |
| **Comfort with watching/addressing mistreatment** | Caregiver reflections on comfort levels watching illustrations of abuse/mistreatment |
| **Motivation to take class** | Caregiver describes why they chose to participate in the program (eg, to help with research, because it looked interesting, because of challenges in their relationship) |
