# Supplementary material for: Qualitative Evaluation of Family Caregivers’ Experiences Participating in Knowledge and Interpersonal Skills to Develop Exemplary Relationships (KINDER): Web-Based Intervention to Improve Relationship Quality
Source: JMIR Form Res. 2023 Aug 22;7:e42561. doi: 10.2196/42561 (PMC10481209; doi:10.2196/42561)
Supplement: Multimedia Appendix 4 [file formative_v7i1e42561_app4.docx]

**Multimedia Appendix 4**

**COnsolidated criteria for REporting Qualitative research**

| **Criteria Number** | **Criteria** | **Page Number** | **Excerpt, if relevant, or explanation** |
| --- | --- | --- | --- |
| **1-3** | **Which author/s conducted the interview or focus group?**  **What were the researcher’s credentials? E.g. PhD, MD**  **What was their occupation at the time of the study?** | 5 | The first author conducted the interview. Her credentials and affiliation are described: *“One-on-one, semi-structured Zoom interviews were administered by the first author, a PhD in Gerontology who was a postdoctoral researcher at the time of data collection.”* |
| **4** | **Was the researcher male or female?** | 5 | Use of “she” pronoun in the sentence, *“She was not involved in administering the intervention . . .”* |
| **5** | **What experience or training did the researcher have?** | 5 | Researcher credentials are described in the following sentence: *“This researcher had graduate-level training in qualitative methods, as well as multiple publications using interview methods.”* |
| **6** | **Was a relationship established prior to study commencement?** | 5 & 6 | The lack of relationship prior to study commencement is described:  **“***She was not involved in administering the intervention to caregivers to prevent the likelihood that caregivers would avoid criticizing the KINDER intervention when speaking with a program facilitator.”*  ***“****Caregivers knew from study recruitment emails and the information sheet that the interviewer was affiliated with a different institute than the institute where the parent study occurred.”* |
| **7-8** | **What did the participants know about the researcher? e.g. personal goals, reasons for doing the research**  **What characteristics were reported about the interviewer/ facilitator? e.g. Bias, assumptions, reasons and interests in the research topic** | N/A | This item is not specifically addressed since participants were not given additional information than what was in the information sheet. The researcher wished to conduct this study for the same reason as those described in the information sheet (i.e., to learn about caregivers’ experiences with KINDER).  Similarly, participants were not aware of anything about the interviewer other than her affiliation and credential listed in the study information sheet. |
| **9** | **What methodological orientation was stated to underpin the study? e.g. grounded theory, discourse analysis, ethnography, phenomenology, content analysis** | 6 | This study used a thematic analysis, known for it’s flexibility and appropriate for this practice-focused/applied research study: *“We used a thematic analysis to analyze the interviews.”* |
| **10** | **How were participants selected? e.g. purposive, convenience, consecutive, snowball** | 5 | The study team used a convenience sample. This is conveyed in the following sentence: *“For this qualitative interview sub-study, participants who completed KINDER were emailed by the program administrator to ask whether they were interested in completing a qualitative interview.”* |
| **11** | **How were participants approached? e.g. face-to-face, telephone, mail, email** | 5 | Participants were contacted by email: *“For this qualitative interview sub-study, participants who completed KINDER were emailed by the program administrator to ask whether they were interested in completing a qualitative interview.”* |
| **12** | **How many participants were in the study?** | 6 | The study included 7 participants: ***“****Of those caregivers, 7 completed the program. The 7 participants who completed the program were invited to complete an interview, and all agreed.”* |
| **13** | **How many people refused to participate or dropped out? Reasons?** | 6 | We had no participants refuse to participate in the qualitative study: *“****“****Of those caregivers, 7 completed the program. The 7 participants who completed the program were invited to complete an interview, and all agreed.”* |
| **14** | **Where was the data collected? e.g. home, clinic, workplace** | 5 | Interviews were collected by Zoom videoconference: *“One-on-one, semi-structured, one-time Zoom interviews.”* |
| **15** | **Was anyone else present besides the participants and researchers?** | 5 | We specify that interviews were conducted one-on-one: “Of those caregivers, 7 completed the program. The 7 participants who completed the program were invited to complete an interview, and all agreed.” |
| **16** | **What are the important characteristics of the sample? e.g. demographic data, date** | 6-7 | Table 2 presents age, gender, race and ethnicity, educational attainment, number of years caregiving, and kin relationship of all study participants. |
| **17** | **Were questions, prompts, guides provided by the authors? Was it pilot tested?** | 6 | The interview guide is provided as a supplementary appendix and Table 1, in the manuscript, describes topics covered: *“Table 1 lists the interview topics, and Supplementary Appendix C provides the interview guide.”*  The guide was not pilot tested. |
| **18** | **Were repeat interviews carried out? If yes, how many?** | 5 | We specify that interviews were one-time (not repeated): *“One-on-one, semi-structured, one-time Zoom interviews.”* |
| **19** | **Did the research use audio or visual recording to collect the data?** | 6 | Interviews were video recorded: ***“****Interviews took approximately 30 minutes to complete, and all were video recorded and transcribed verbatim.”* |
| **20** | **Were field notes made during and/or after the interview or focus group?** | N/A | Notes were not made during or after the interview, so this is not noted in the manuscript. |
| **21** | **What was the duration of the inter views or focus group?** | 6 | Interviews took 30 minutes to complete: *“Interviews took approximately 30 minutes to complete, and all were video recorded and transcribed verbatim.”* |
| **22** | **Was data saturation discussed?** | N/A | Given the small available sample size, the authors did not attempt to reach data saturation. |
| **23** | **Were transcripts returned to participants for comment and/or comment** | N/A | Transcripts were not returned to participants, and thus we do not note this in the manuscript. |
| **24** | **How many data coders coded the data?** | 6 | Data was coded by two coders and a third researcher reviewed consistency of codes: *“Transcripts were analyzed by two independent coders. . . . Prior to conducting a second round of coding, a third researcher reviewed all code categories to ensure consistent application of codes to text excerpts to enhance the trustworthiness of findings [23].”* |
| **25** | **Did authors provide a description of the coding tree?** | 6 | A coding tree is provided within an appendix: *“An abbreviated codebook can be found in Multimedia Appendix B.”* |
| **26** | **Were themes identified in advance or derived from the data?** | 6 | Themes were not identified in advance. We believe our described inductive and deductive approach to coding conveys this: ***“****Codes were developed to address topics discussed in the interview guide, though coders also considered inductively-derived codes.”* |
| **27** | **What software, if applicable, was used to manage the data?** | 6 | The researchers used NVivo 12: *“Analyses were conducted in NVivo 12.”* |
| **28** | **Did participants provide feedback on the findings?** | N/A | Participants did not provide feedback on findings, and thus this was not described in the manuscript. |
| **29** | **Were participant quotations presented to illustrate the themes/findings? Was each quotation identified? e.g. participant number** | 7-11 | Participant quotations were used to illustrate themes/findings. Each quotation is identified by participant number. |
| **30** | **Was there consistency between the data presented and the findings?** | N/A | Findings and data are consistent. This is supported by two rounds of coding by independent coders, followed by review by a third coder. |
| **31** | **Were major themes clearly presented in the findings?** | 7-10 | Major themes are clearly delineated in the Results section, wherein subheadings are used to describe each theme. |
| **32** | **Is there a description of diverse cases or discussion of minor themes?** | Page 9; Paged 10-11 | **Pages 10 to 11:** The authors included minor themes that did not fit with main themes, such as feedback that the intervention would be most suited for newer caregivers, that some caregivers felt there was too much content, and some concerns about the quality of materials.  **Page 9:** The authors also describe a negative case of a caregiver who related that she was felt uncomfortable with portrayals of caregiver anger and mistreatment: “Still, one caregiver did report feeling somewhat uncomfortable with portrayals of mistreatment. ‘The anger, I think, made me feel uneasy.’” |
